# Meningeal Lymphatics: An Immune Gateway for the Central Nervous System

**DOI:** 10.3390/cells10123385

**Published:** 2021-12-01

**Authors:** Gabriel A. Tavares, Antoine Louveau

**Affiliations:** 1 Department of Neurosciences, Lerner Research Institute, Cleveland Clinic, Cleveland, OH 44195, USA; TAVAREG@ccf.org; 2 Department of Molecular Medicine, Cleveland Clinic College of Medicine, Case Western Reserve University, Cleveland, OH 44195, USA

**Keywords:** meningeal lymphatics, immune cells, neurological disorders, brain pathologies

## Abstract

The recent (re)discovery of the meningeal lymphatic system has opened new theories as to how immune cells traffic and interact with the central nervous system (CNS). While evidence is accumulating on the contribution of the meningeal lymphatic system in both homeostatic and disease conditions, a lot remains unknown about the mechanisms that allow for interaction between the meningeal lymphatic system and immune cells. In this review, we synthesize the knowledge about the lymphatic immune interaction in the CNS and highlight the important questions that remain to be answered.

## 1. Introduction

The (re)discovery of the meningeal lymphatics reveals new concepts about central nervous system (CNS) drainage and immune cell trafficking that has prompted a review of immune cell privilege in the CNS [1]. The meningeal lymphatics play an important role in this context by both draining the fluid within the subarachnoid spaces and allowing immune cell recirculation, and possibly being the main route of communication between the brain and the periphery [2]. In this review, we highlight the contribution and mechanisms of immune cell trafficking through the meningeal lymphatics in neurological disorders and emphasize the future of research on the topic.

## 2. Meningeal Lymphatics: Concepts, Development, and Extension of the Network

**Figure 1:**
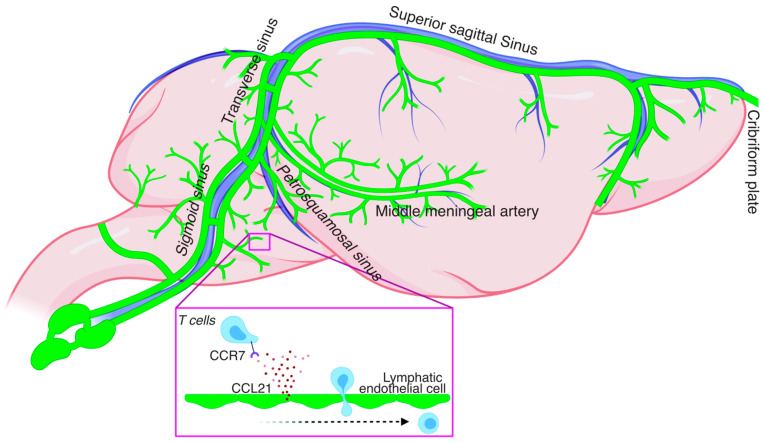
Scheme of rodent meningeal lymphatic network and CCR7-dependent T cell entry into the meningeal lymphatic vessel.

The rodent meningeal lymphatics vessels (mLVs) are a network of lymphatic vasculature extending in the entirety of the meningeal compartment starting most rostrally from the cribirform plate in front of the olfactory bulbs all the way to the lumbar regions of the spinal cord [3,4,5,6] (Figure 1). The mLVs are primarily located in the outmost layer of the meninges called the dura and are physically separated from the cerebrospinal fluid (CSF) by the arachnoid barrier. Originally described by Paolo Mascagni in the 1600s [7], they were only recently functionally characterized in rodents [3,8], zebrafish [9,10], primates, and humans [8,11].

Elegant studies have used transgenic mice to investigate how the mLVs develop in mammals [12,13,14]. Interestingly, mLVs develop during the postnatal period differing from other tissues where the lymphatics develop earlier in development [12,13,14]. Using Prox1-GFP reporter mice and immunostaining against lymphatic markers, such as Lyve-1 and Prox1, it has been shown that primary meningeal lymphatic structures are present at the base of the skull shortly after birth at postnatal day 1 (P1) [12]. Similarly, a lymphatic network is observed at the cribriform plate as soon as P2 [12]. Between P4 and P8, the sprouts from the base of the skull reach the medial meningeal artery and the surroundings of the foramen magnum and cisterna magna. By P16, the sprouts fully extend through the transverse sinus and the confluence of the sinus. Next from P20 to P28, the mLVs reach the full extension of the superior sagittal sinus, medial meningeal artery, and rostral rhinal vein [12]. Finally, by P28, the mLVs reach full functionality and extension, and surround the main dural vessels and sinuses [12].

Even though mLVs develop later than the lymphatics of peripheral tissues, mLV development is similarly dependent on the VEGF-C (Vascular Endothelial Growth Factor-C)–VEGFR-3 (Vascular Endothelial Growth Factor Receptor-3) pathway to drive lymphangiogenesis [12]. It has been shown that the agonism or antagonism of the VEGF-C–VEGFR-3 pathway can, respectively, stimulate or impair meningeal lymphangiogenesis during the developmental period. It is interesting to note that the mLVs maintain their requirement for VEGF-C-VEGFR3 signaling during adulthood [12], a feature not shared by peripheral lymphatic vessels. Upon maturation, the meningeal lymphatics become functional and drain to the cervical lymph nodes [3,8,13]. Using tracers administered within the CSF, it has been shown that its lymphatic drainage is performed mainly by two routes: the nasal mucosa lymphatics that drain to the superficial cervical lymph nodes (scLN) and by the dural lymphatics that drain to the deep cervical lymph nodes (dcLN) [3,4,6,8,15].

What makes the initial characterization of this network so fascinating is the possibility of mLVs acting as an avenue for trafficking immune cells and molecules out of the CNS. Indeed, immune cells are observed within the lumen of mLVs, suggesting that the meningeal lymphatics may play an important role in brain immune surveillance [4,8,15]. Major gaps in knowledge still remain about how mLVs interact with and control cell trafficking in and out the brain in either physiological or pathological conditions, and if mLVs could be used as a route for therapeutic intervention in several neural disorders. Consequently, we will discuss the current knowledge regarding immune cell trafficking within the CNS in both health and disease and the role of meningeal lymphatics in this process. Hopefully this review will help us to drive future directions for research regarding the management of CNS diseases in the light of the meningeal lymphatics.

## 3. Immune Cell Trafficking under Homeostatic Conditions

The blood–brain barrier (BBB) restricts the access of cells or molecules coming from the periphery into the CNS parenchyma [16]. However, immune cells can access the CNS parenchyma (predominantly during inflammation from infection) via the meningeal spaces [17,18]. These cells can be retained within the CSF-containing meningeal spaces in the absence of inflammation and/or infection thus acting to surveil the CNS [19,20].

Diverse types of immune cells can be found within the meninges and the meningeal spaces, specifically in the subarachnoid space (SAS). Recent single-cell transcriptomic studies have started to highlight the richness and unique origin of the immune cells populating the meningeal compartment [21,22,23,24]. Multiple immune cell types, particularly T lymphocytes (CD4), have been demonstrated to be essential for the maintenance of higher brain function [25,26,27,28,29]. Therefore, despite not being directly present in the brain parenchyma, the immune cells present within the CSF of the SAS and the meningeal spaces can exert immune and non-immune functions that will alter neuronal behavior [27].

Homeostatic regulation of the meningeal immune compartment is only starting to be explored. The mLVs appear to play a role in the maintenance of some meningeal immune cells [15]. Indeed, impairment of the mLVs either systemically or locally results in the accumulation of T lymphocytes in the meninges [15]. One explanation of these observations is that the lack of functional lymphatics is disrupting the natural dynamics of T cell circulation out of the meninges leading to T cell accumulation that is normally drained under homeostatic conditions. Similar to the periphery, the chemokine receptor 7 (CCR7)-chemokine ligand 21 (CCL21) pathway appears to be essential for drainage via the mLVs (Figure 1). Intrathecal injection of CCR7 deficient T cells or dendritic cells results in their accumulation in the CNS and failure to drain into the cervical lymph nodes thus emphasizing the dependence of CCR7-CCL21 signaling in immune cell drainage [15,30]. The dynamic circulation of other immune cells such as macrophages and monocytes and the implication of the mLVs in this process remains unaddressed at this point.

## 4. Immune Cells Trafficking in Pathological Conditions

### 4.1. Multiple Sclerosis (MS) and Autoimmunity

MS pathophysiology involves localized inflammation sites with characteristic local demyelination, astrogliosis, and a marked infiltration of active T cells [31,32]. The origin of the inflammation in MS is still a matter of discussion, as it is still not clear if the inflammatory process begins inside or outside the CNS [33]. However, one of the most accepted mechanisms for MS pathophysiology is an autoimmune process. For that reason, one of the most used experimental models of MS is Experimental Acute Encephalomyelitis (EAE), in which animals are peripherally exposed to the myelin antigen that leads to a progressive demyelination and characteristics symptoms of MS [33].

Regarding immune cell trafficking in MS/EAE, it is postulated that the BBB is dysfunctional, and this allows for the abnormal infiltration of encephalitogenic T cells into the brain parenchyma through the perivascular spaces of the brain vessels [34]. An interesting study shows that the leptomeninges play a key role as a checkpoint for T cells by determining which ones are allowed to infiltrate the brain parenchyma [17]. Antigen-presenting cells (APC) within the meningeal spaces present antigens that causes local T cell reactivation. The encephalitogenic T cells then infiltrate the parenchyma and attack the myelin leading to CNS lesions. One of the main APCs observed in this context is the B cell which is enriched within the meningeal compartment and CSF, even forming clusters and secondary lymphoid structures [35]. For a long time, dysfunctions in the blood–cerebrospinal fluid barrier (BCSFB) were believed to allow the massive infiltration of lymphoid cells within the CSF [36].

However, recent studies have shown that the meningeal lymphatics play a key role in immune cell trafficking in the EAE context. Louveau et al. have shown that the dorsal mLVs represent an essential route for the drainage of immune cells and molecules to the cervical lymph nodes. In the same study, it was shown that the meningeal lymphatics are even a possible target for management of the disease progression because the ablation of the mLVs decreases the encephalitogenic phenotype of antigen-specific T cells and leads to improvement of the clinical symptoms in EAE [15]. Additionally, lymphangiogenesis is observed in the lymphatic vessels of the cribriform plate during EAE in a VEGF-C-VEGFR3-dependent manner [6]; furthermore, these vessels are implicated in the drainage of CNS dendritic cells to the cervical lymph nodes in order to maintain T cell activation and antigens that lead to increased proliferation of reactive T cells in the draining lymph nodes [6]. In this context of MS/EAE, the meningeal lymphatics appear to be an active modulator of immune cell trafficking in and out the brain. Overall, these studies highlight that the mLVs contribute to the maintenance of the immune response in the context of EAE. Understanding if it is through its draining function of CSF constituents (myelin?) or immune cells (APC), or through an as yet undescribed mechanism, remains to be investigated.

### 4.2. Brain Injury

In traumatic brain injury (TBI), it is well documented that one of the main events contributing to neural damage post-injury is the brain infiltration of immune cells, specifically T cells [37,38,39,40], and T cell infiltration is one of the main factors responsible for chronic neurological impairments following TBI [38]. This T cell infiltration to the lesion sites has a marked temporal activity after the injury. Generally, a robust T cell infiltration occurs immediately after the trauma and reaches a peak within 3 days after injury [40]. One month after the injury, there is a second wave of immune cell recruitment to the lesion site that is characteristic of a late immune response and persists chronically [39]. Besides TBI, T cell infiltration is also observed in the context of intracerebral hemorrhage. Zhang et al. demonstrated a temporal pattern for this infiltration, increasing 1 day after the injury and peaking 5 days later. Furthermore, the same study reported that the T cell infiltration in the hemorrhage context leads to BBB injury and increasing BBB leakage [41].

However, the role of the meningeal lymphatics in immune cell dynamics remains unknown post hemorrhagic insult. The mLVs have been shown to drain erythrocytes produced by the brain hemorrhage [42,43]. Moreover, it has been shown that 1 h post brain hemorrhage the mLVs increase in diameter suggesting that blood components may affect meningeal lymphatic morphology.

In addition to the drainage of blood products after injury, a few studies have suggested a potential role of the meningeal lymphatics in immune cell circulation after brain injury. Bolte et al. (2020) demonstrated that traumatic brain injuries lead to impairment of meningeal lymphatics up to a month post injury. They showed that pre-existing impaired function of the meningeal lymphatics is associated with worse outcomes after TBI such as increased neuroinflammation and worse cognitive outcomes [44], a process previously linked to the extent of the immune response to the TBI. Yanev et al. used two different models of brain injury: photothrombosis approach and the transient middle cerebral artery occlusion to study the involvement of the mLVs [45]. They showed that depending on the injury model the mLVs may or may not undergo lymphangiogenesis. Additionally, transgenic mice with mLVs hypoplasia presented with worst stroke outcomes depending on the injury model [45]. These studies highlight how the mLVs may respond and influence injuries differently, depending on the model and microenvironment associated with it. Additionally, in K14-VEGFR3-deficient mice, a mouse lacking meningeal lymphatics (among other peripheral lymphatics), the infiltration of T cells to the brain parenchyma is reduced. This shows that the meningeal lymphatics may be an important route for the immune cell trafficking after brain injury [46] and may be caused by a reduction of drainage of factors stimulating T cell activation in the periphery. Like in MS, while all these studies demonstrate that (1) a pathological CNS can be associated with change in mLVs morphology and function and (2) modulating the mLVs can impact disease pathology, more studies are necessary to better understand the molecular mechanisms involving the mLVs, particularly regarding their interaction with immune cells.

### 4.3. Brain Tumor

Recent data show that the meningeal lymphatics are important actors in regulating the drainage and immune response of brain tumors. Indeed, increased functionality of the mLVs has been shown to ameliorate the tumor response. In a mouse model of glioblastoma, ectopic administration of VEGF-C leads to increased activation of CD8+ T-cells in the dCLNs and improved their migration to the tumor site leading to slower tumor growth [47]. Additionally, it has been demonstrated through RNAseq that genes associated with lymphatic remodeling and fluid drainage, as well as inflammatory and immune responses, are increased in the dorsal mLVs in response to tumoral activity, suggesting an active remodeling of the mLVs by the tumor [48]. The tumor interstitial fluid and dendritic cell drainage to the dCLNs is impaired in mice lacking dorsal MLVs, and the opposite is observed in induced lymphangiogenesis [48]. Moreover, the integrity of the dMLVs appears to be associated with a better response to the conventional tumor treatments in the case of striatal tumor models [48].

An interesting study sought to evaluate the preventive versus therapeutic treatment with VEGF-C in tumor models [49]. They found that the preventive administration of VEGF-C inhibits tumor growth and increases survival rates in mice [49]. Additionally, treatment administered after tumor implantation that combines VEGF-C and conventional immunotherapy impairs tumor growth [49]. These results are proposed to be due to the increased activation of T cells in the cervical lymph nodes that lead to an improved immune response against the tumors [49].

Together, this evidence supports the notion that an increase in mLVs function results in a better and durable immune response/memory against brain tumor. These studies provide a great backbone to try to better understand how tumor immunogenicity is regulated in vivo and how the mLVs may contribute to the immune cell response in tumor models.

### 4.4. Neurodegeneration

A couple of studies have sought to investigate the involvement of the meningeal lymphatics with the pathophysiology of neurodegenerative diseases such as Alzheimer’s disease (AD) and Parkinson’s disease (PD).

In AD, it is well accepted that the mLVs are important for the drainage of amyloid beta (Aβ) and tau [50,51,52,53]. Da Mesquita et al. have shown that the impairment of the mLVs leads to increased accumulation of Aβ plaques and worse cognitive performance. On the other hand, improving meningeal lymphatics function in AD mice leads to a better clearance of Aβ and partially rescues cognitive impairments [54]. Furthermore, it was shown that a functional meningeal lymphatic network is essential for a better response to passive immune therapy with anti-Aβ [52]. These data demonstrated in mice that a lack of functional mLVs may not only be involved in the physiopathology of AD, but also may influence therapeutic strategies. Besides the involvement of the mLVs in the clearance of Aβ, Patel et al. (2019) demonstrated, in mice, that lacking functional mLVs impairs tau drainage and overall shows that mLVs are involved in both types of AD pathology. Reinforcing the essential role of the mLVs in AD, in APP/SS1 mice the ligation of the dCLNs (which drastically reduces drainage and CSF homeostasis) increased the AD-like phenotype in these mice with more Aβ accumulation and cognitive impairment [55].

Despite the lack of data specifically about immune cell circulation in AD brains, we know that the aging process promotes a decrease in CCR7 on meningeal T cells that is proposed to impair the egress of the cells from the meninges and increase the effector and regulatory T cell profile. The deletion of CCR7 in 5xFAD (a mouse model of AD) mice leads to accelerated cognitive decline and Aβ accumulation that is improved by anti-CD25 treatment (which depletes both activated and regulatory T cells) in old mice [51]. These data support the idea that the aging process leads to the accumulation of regulatory T cells that contributes to the AD pathology and may act through the modulation of immune cell recirculation via the meningeal lymphatic system.

Regarding PD, the literature is more scarce; however, it was recently demonstrated that patients with idiopathic forms of PD show a decrease in mLV functionality [56]. It has been shown that A53T (a model of PD with expression of a mutated form of human α-synuclein) mice have impaired CSF homeostasis with increased accumulation of α-synuclein within the perivascular spaces [57]. Interestingly, the ligation of the dCLN in these mice leads to an even worse accumulation of α-synuclein, glial activation, inflammation, dopaminergic neuronal loss, and motor deficits [57]. These results are similar to the ones published by Ding et al. that demonstrate mice undergoing α-synuclein administration present worse pathological and behavioral outcomes after ligation of the mLV. In addition, Ding et al. showed that the mice injected with α-synuclein presented delayed mLVs drainage, loss of tight junctions among meningeal lymphatics endothelial cells, and meningeal inflammation [56].

To date, no studies have specifically addressed how immune cell recirculation through the mLVs contributes to disease pathophysiology. However, in both AD and PD circumstantial evidence suggests that immune cell drainage will be impaired in disease conditions and potentially influence disease progression.

## 5. Burning Questions about Immune Cell Trafficking through the Meningeal Lymphatic System

### 5.1. Origin of Immune Cells Draining through the Meningeal Lymphatics?

While we have considerable indirect evidence that meningeal and CSF injected immune cells can enter the meningeal lymphatics to reach the cervical lymph nodes, we currently have limited information about such a path in physiological conditions. Histological analyses under naïve and inflamed environments demonstrated the presence of intralymphatic immune cells [4,6,8,15], however the origin of such cells remains unknown. Are these cells solely originating from the outer layers of the meninges? The CSF? Or could they be coming from the brain parenchyma? Technical limitations of intracerebral/intrathecal injections have impeded drawing definitive conclusions; furthermore, injections create some degree (even if limited) of physical disturbance of the fluid dynamics in the CNS that may generate artifacts. Moreover, some recent studies have questioned the effect of perfusion and ex vivo analysis on previously observed CSF dynamics [58], but state-of-the-art-controlled live imaging validated the natural diffusion of CSF into the perivascular spaces of the brain parenchyma [59]. The brain is a unique environment for immune cells. Under physiological conditions, most immune cells (except for microglia) are sequestered to the borders of the brain either the meninges, choroid plexus, or perivascular spaces [60]. Is the brain repelling the infiltration of immune cells under normal conditions? Or rather, are the borders actively retaining cells within these confined compartments? If so, when immune cells (T cells, dendritic cells, B cells, monocytes, and neutrophils) have infiltrated the brain under neuroinflammatory conditions, can and will they exit? Are all types of immune cells going to display different dynamics of migration? One such suggested path of migration out of the brain is the rostral migratory stream [61]. Dendritic cells migrating through the rostral migratory stream could exit the brain at the level of the olfactory bulbs and reach the lymphatics at the levels of the cribriform plate [6]. On the other side, the brain is thought to mostly be a detrimental environment for immune cells to survive, primarily through high levels of Fas signaling [62]. Future research on the migration dynamics of immune cells in and out of the different compartments of the CNS will be necessary to decipher the origin of immune cells migrating through the meningeal lymphatics.

### 5.2. Accessibility of the Meningeal Lymphatics to Immune Cells?

The description of the meningeal lymphatics, whether located on the dorsal or basal part of the skull, demonstrate that the majority of the vessels are located within the dura, the most outer layer of the meninges [4,15]. This specific location raises the question of accessibility of the CSF and its constituents to the meningeal lymphatics, particularly because the arachnoid layer has been described to be impermeable. Nonetheless, experimental evidence demonstrates that intrathecal injection of tracers or immune cells results in their uptake by the meningeal lymphatic system, but the path and mechanisms for this is completely unknown. Studies have suggested that the meningeal lymphatics may extend sprouts through the arachnoid layers to facilitate CSF uptake [15] or that the structure of the dura itself is adapted to allow drainage [4]. However, none of them provide the fundamental evidence to demonstrate how immune cells of the CSF can migrate and enter the lymphatic system. Can immune cells alter the tight junction architecture of the arachnoid to facilitate their migration? Do the meningeal layers, whether arachnoid or dura, present some regionality and lose their impermeability properties in very discrete regions to allow drainage into the lymphatics? The very localized patterning of ovalbumin uptake in the meninges upon intrathecal injection does suggest some localized and discrete diffusion of the CSF into the upper meningeal layers.

### 5.3. Are Meningeal Lymphatic Endothelial Cells Altering Immune Cell Phenotypes?

Immune cells can be found within the meningeal lymphatics under both physiological and inflammatory conditions. Very little is known about the interaction of these immune cells with the lymphatic endothelial cells. Unsurprisingly, the CCR7-CCL21 pathway appears to be necessary for drainage of immune cells into the cervical lymph nodes [15,51]. New studies are demonstrating that in the skin, interaction of immune cells with lymphatic endothelial cells is not limited to facilitating entry, but rather continues once into the initial lymphatics [63,64]. Does a similar phenomenon arise in the meningeal lymphatics? How does this interaction impact the phenotype and function of the draining immune cells? Development of in vivo imaging approaches to study the localized interaction of immune cells with the meningeal lymphatic endothelial cells will be essential to address these questions.

### 5.4. CSF Homeostasis vs. Immune Cell Trafficking, Who Is to Blame in Neurological Disorders?

The meningeal lymphatic system appears to be central for the migration and drainage of soluble CSF constituents and immune cells. Moreover, a functioning meningeal lymphatic network is central for the maintenance of CSF dynamics in general [50]. Currently available tools are manipulating the meningeal lymphatics using their main growth factor pathway, VEGF-C-VEGFR3. While extremely useful, such approaches fail to allow the discrimination between CSF homeostasis and immune cell trafficking. Accumulating evidence is showing the meningeal lymphatics may have both beneficial and detrimental functions depending on the neurological disorder. Therefore, completely ablating or boosting meningeal lymphatic function may induce unwanted side effects. Deciphering the contribution of CSF homeostasis and immune cell trafficking in different neurological conditions may help refine the development of therapeutic strategies aimed at meningeal lymphatic function.

### 5.5. Which Pathways Are Immune Cells Using to Migrate through the Meningeal Lymphatics?

The presence of immune cells within the mLVs has been observed both under normal and pathologic conditions, whether they are T cells [4,15], B cells [21], dendritic cells [6,15], or erythrocytes [42]. The mechanisms that allow the entry and migration of such immune cells within the meningeal lymphatic remain poorly characterized. Unsurprisingly, the CCR7-CCL21 pathway has been suggested to be necessary for the migration of T cells and dendritic cells via the mLVs [15]. Yet, a formal, in vivo demonstration of the physiological requirement of CCR7 expression by immune cells remains to be demonstrated. While loss of CCR7 reduces the drainage of T cells and dendritic cells, it is not fully abolished, demonstrating the requirement of other pathways. Multiple ligand:receptor pairs expressed in lymphatic endothelial cells and immune cells have been shown to mediate trafficking through the lymphatic vasculature including Lyve1: Hyaluronic Acid [65], ICAM1:beta 2 integrins [66,67,68], CXCR4:CXCL12 [69], S1P:S1PR [70,71,72], and CX3CL1:CX3CR1 [73], which are among such molecules expressed by lymphatic endothelial cells and immune cells to allow draining [74]. More studies are required to identify if similar pathways, or other mechanisms are controlling the migration of meningeal immune cells to and through the mLVs.

## 6. Conclusions

In conclusion, despite accumulating circumstantial evidence that immune cells can traffic through the meningeal lymphatic system under normal and pathological conditions, we still only know very little about the mechanisms governing such trafficking and the functional role of the meningeal lymphatics in regulating these immune cells. Further research is necessary to characterize and harness the lymphatic immune interaction to treat neurological disorders.
